# Shrub cover homogenizes small mammals’ activity and perceived predation risk

**DOI:** 10.1038/s41598-019-53071-y

**Published:** 2019-11-14

**Authors:** Anne A. Loggins, Adrian M. Shrader, Ara Monadjem, Robert A. McCleery

**Affiliations:** 1 School of Natural Resources and the Environment, University of Florida, Gainesville, Florida USA; 2 Mammal Research Institute, Department of Zoology & Entomology, University of Pretoria, Private Bag 20, Hatfield, 0028 Pretoria South Africa; 3 Department of Biological Sciences, University of Eswatini, Private Bag 4, Kwaluseni, Eswatini; 4 Department of Wildlife Ecology and Conservation, University of Florida, Gainesville, Florida USA

**Keywords:** Conservation biology, Behavioural ecology

## Abstract

Altered disturbance regimes, increasing atmospheric CO_2_, and other processes have increased woody cover and homogenized vegetation in savannas across the planet. African savannas with extensive versus minimal woody cover often have vastly different animal communities. However, we lack a clear mechanistic understanding of why animal communities are changing with vegetation structure. Our goal for this study was to understand how vegetation structure in an African savanna shaped the perceived predation risk of small mammals, hence affecting their activity. Using a reciprocal measure of standard giving-up-densities, amount of food eaten, we found sharp declines in rodents’ perceived predation risk and increased rodent activity underneath shrub cover. This response was consistent across species; however, species showed subtle differences in their responses to grassy vegetation. Our findings suggest that areas of minimal or extensive shrub cover (shrub encroachment) may be homogenizing rodents’ perceptions of predation risk and thus shaping their use of space.

## Introduction

Savannas can be characterized by the competition between grass and woody vegetation. The ratio of woody to grass cover is highly dynamic and can change rapidly over time and space^1–3^. However, these dynamics are increasingly altered by anthropogenic factors that favor one component over the other. Altered disturbance regimes (i.e. fire suppression, cattle grazing, loss of native browsers, predator removal), increasing atmospheric CO_2_, and other processes have increased woody cover in savannas around the globe and particularly in Africa^4–6^. In contrast, the removal of big trees, firewood harvesting, and extensive browsing by spatially-confined herbivores can all cause the broad-scale reduction of woody cover in savannas^7–10^.

There is growing evidence that savannas with minimal woody or grass cover have different animal communities and often show reductions in the richness and diversity of mammals compared with more heterogeneous savannas^11–15^. However, we lack a clear mechanistic understanding of why animal communities are changing with vegetation structure. While changes in the amount of, and access to, food might provide one explanation^11,16–18^, it appears insufficient to explain an animal’s use of environments with different types of vegetation structure^18–20^. Accordingly, it is possible that changes in vegetation may change an animal’s perceived predation risk by increasing a potentially risky structure^18,21,22^. In fact, fine-scale changes to the vegetation structure have been shown to alter the fear levels of prey, regardless of the abundance of predators^21,23^, and influence prey’s perceived predation risk more than actual predator cues^24^.

Many smaller vertebrates preferentially forage under vegetative cover, where it is more difficult for predators to detect them, avoiding areas with sparse cover or greater distances between refuges^21,25–28^. Alternatively, other species prioritize foraging in areas with increased sightlines to spot predators earlier, thereby increasing their chances of escape (e.g.^23,29,30^). Avoiding patches or increasing vigilance in risky patches creates tradeoffs with other fitness-improving activities such as reproduction and foraging^31^. Accordingly, animals must minimize their risk of predation while maximizing foraging and reproductive opportunities. Under different levels of predation risk, these tradeoffs can produce changes in individual survival and fitness^31^, as well as broad-scale shifts in the distributions of animal species and communities^27,32,33^.

Most large African herbivores (>20 kg) appear to avoid areas of dense shrubby vegetation^13,18^ where they have an elevated perception of predation risk (but see^34,35^) from reduced visibility and mobility^18–20,34^. However, there has been minimal effort to understand if small mammals’ (<1 kg) differential use of woody and grassy cover in savanna systems^27,28^ is a function of variation in the perceived predation risk^36^. Small mammals are ecologically important to savannas as seed predators, ecosystem engineers, nutrient cyclers, and prey species^37^. In savannas^38,39^ and many other systems^40–43^, safety for small mammals is correlated with some measure of vegetation density such as shrub cover or grass height. With anthropogenic forces altering the ratio of grass and woody components in savannas, perceptions of fear may facilitate shifts in small mammal communities. For example, potentially elevated levels of fear may provide a mechanistic explanation for the pattern of depauperate rodent communities in savannas with minimal cover, but where ample food resources persist^12,22,44^. One way to initiate an understanding of the factors that influence small mammals’ perceptions of fear is to investigate their behavior on the fine scales at which they conduct most of their daily activities such as foraging for food^45^.

Our goal for this study was to understand how vegetation structure influences perceived predation risk and the activity (i.e. time spent foraging at a location) of different species comprising a small mammal community in a shrub encroached savanna. Our objectives were to understand: 1)how shrubs (i.e. woody vegetation <3 m tall) alter small mammals’ perceptions of fear; 2)how variation in the structure of grassy environments influences small mammals’ activity and perceived predation risk; and 3) discuss the broader implications of small mammals’ variation in activity and perceived predation risk around shrubs. Due to protection from avian and mammalian predators we predicted that the proximity to a shrub would decrease perceived risks and hence increase the activity of small mammals under high shrub cover^38,39,41–43^(Online Resources 1). We also predicted that shorter grass (<40 cm) and reduced horizontal visual obstruction would increase perceived risk and decrease activity levels^28,38,40^.

## Materials and Methods

When feeding, animals will remain in a resource patch until the nutritional benefits of feeding in that patch no longer outweigh the costs of feeding in the patch^46–48^. To tease apart the influence of the different costs associated with feeding in a patch (i.e. metabolic, predation, and missed opportunity costs), Brown^47^ introduced the “Giving-up Density” (GUD) methodology. By placing identical artifical foraging patches in a landscape, the opportunity costs of foraging are equalized across the environment, and hence the relative predation costs will be proportional to the harvesting rate at each foraging patch. Less food remaining in a foraging patch suggests a “safer” location where the animal perceives lower costs to continuing foraging in order to maximize nutritional intake. By comparing the GUDs obtained in artificial food patches in different habitats or microhabitats, we can better understand why animals use the landscape in the way that they do^27^.

We used GUDs^47^ to assess small mammals’ perceived predation risk across a fine-scale gradient of shrub and grass cover in the savannas of Eswatini. Additionally, by using camera traps to monitor foraging patches, we quantified the activity levels of individual species and the small mammal community as a whole.

### Study sites

**Figure 1:**
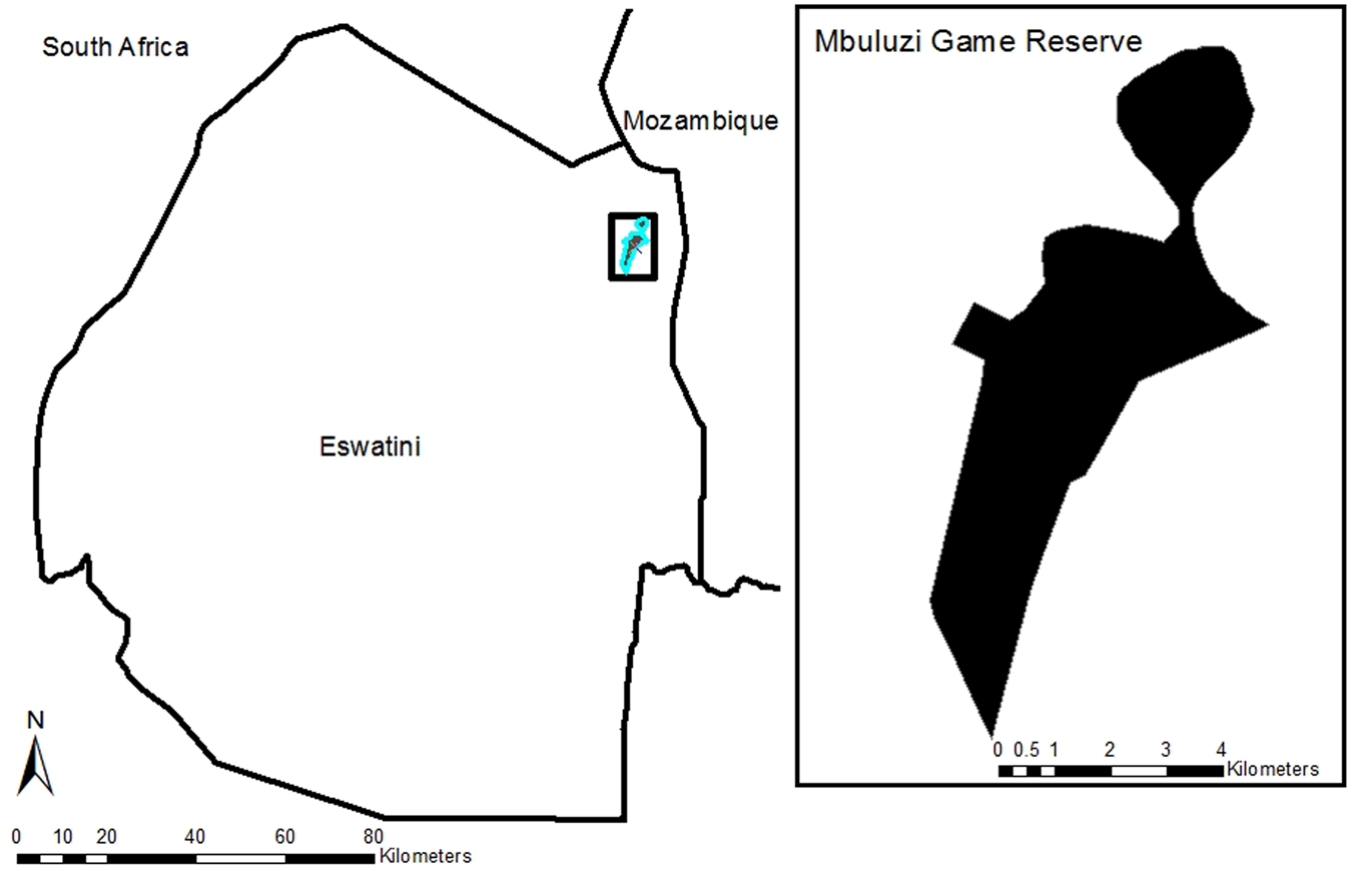
Location of the study site, the Mbuluzi Game Reserve, within the kingdom of Eswatini.

We conducted GUD experiments in Mbuluzi Game Reserve (30 km²) in Eswatini (26.1564°S, 31.9824°E, Fig. 1). Located in the lowveld region adjacent to the Lubombo Mountains on basaltic soils, this protected area is currently managed for wildlife conservation and ecotourism with minimal resource extraction (e.g. wood and grass harvest). This area has a subtropical climate and a unimodal rainfall pattern with the wet season during October-March (when 75% of the annual rainfall is received) and the dry season during April-September (25% of the annual rainfall)^49^. Yearly precipitation in the region typically ranges from 500–750 mm^50^. *Senegalia nigrescens* and *Sclerocarya birrea* were the dominant tree species in the savannas. The dominant grass species included *Themeda triandra* and *Panicum maximum*, while *Dichrostachys cinerea* was the dominant shrub. This region, and our site, has seen a drastic increase in shrub cover over the last the 70 years, from 2% to >40% at the time of the study^51,52^.

The most common large mammals on the study sites included impala (*Aepyceros melampus*), warthog (*Phacochoerus africanus*), giraffe (*Giraffa camelopardalis*), zebra (*Equus quagga*), blue wildebeest (*Connochaetes taurinus*), kudu (*Tragelaphus strepsiceros*), and nyala (*Tragelaphus angasii*)^13^. Elephants (*Loxodonta africana*), white rhinoceros (*Ceratotherium simum*), and buffalo (*Syncerus caffer*) were extirpated from the region around 1920^53,54^. Small mammal predators on the site included jackals (*Canis mesomelas*, *C. adustus*), serval (*Leptailurus serval*), and large-spotted genet (*Genetta maculata*), as well as snakes (e.g. black mamba [*Dendroaspis polylepis*]) and predatory birds such as the spotted eagle owl (*Bubo africanus*), and the lizard buzzard (*Kaupifalco monogrammicus*).

### Small mammal species

At least 10 small mammal species in the orders Rodentia, Eulipotyphla, and Macroscelidea, ranging in size from 5–100 g, were present at Mbuluzi Game Reserve. These species occupy vegetation communities ranging from open grasslands, mixed woodlands, cultivated farmlands, and rocky terrain^55–58^. Rodent species are granivorous or omnivorous with seasonal shifts in diet^27,57,59,60^ (Online Resources 2). All species were nocturnal except the single-striped grass mouse (*Lemniscomys rosalia*), which is largely crepuscular^57^.

### Data collection: estimating perceived predation risk

We conducted GUD experiments in the austral winter from May-August 2016 when the rodents are most abundant in this region^61^. We placed five artificial foraging patches comprising a circular plastic tray (30 cm diameter, 2 cm high) in a transect line as follows: 1 m within a shrub (*Shrub*); at the shrub/grass interface (*Edge*; approximately 50% shrub coverage); and 50 cm, 1 m, and 3 m into an open grassy area (*Grass*: *50 cm*, *1 m*, *3 m*; Online Resource 3). We defined a “shrub” to be a connected cluster of woody plants with a volume of at least 2 m³, and 1–3 m in height. Several shrub species were present in our study area, but where possible we selected *Dichrostachys cinerea* bushes for our experiments (≈70% of transects) as this was the most common encroaching woody species at the site. The last patch in the grassy area, which marked the end of the transect, was placed 3 m from the focal shrub and a minimum of 3 m from other shrub clusters in all directions. We placed transects >50 m apart and sampled a total of 15 transects. We did not place the foraging patches near large (>5 m tall) trees to avoid the confounding effects of canopy cover and reduce the influence of avian seed consumers.

Before collecting GUD data, we habituated the rodents to the artificial patches by putting them out with millet seeds for at least 3 days. Once we saw evidence that rodents were using the patches (removed food, droppings, seed fragments, or shells), we mixed 600 g of sand with 40 ml (33 g) of hulled millet seeds per foraging patch. We determined the appropriate amount of seed and sand to use during a trial period to ensure that the animals were not consuming all the seeds in a tray^27,28^. Mixing the food with sand mimics a natural foraging scenario where there are associated costs with time spent foraging^47^. To ensure that the rodents were familiar with the sand and seed mixture, we put these artificial foraging patches out for a minimum of 2 days before data collection began. We captured the foraging of nocturnal and diurnal small mammals by sifting out the remaining seeds once every 24 hours at sundown. To determine the GUD, we measured the volume of seeds remaining, to the nearest ml, in each foraging patch each day. We then refilled the patches with 600 g of sand and 40 ml (33 g) of hulled millet seeds. We monitored the foraging patches over the course of 5–7 days (24-hour periods) and generated count data by recording the number of mls removed to the nearest whole ml (0–40 ml, *Food Eaten*).

To remove the confounding influences of non-target species^21,27,62^ and estimate the time of visits by the entire community and individual species, we attached a Reconyx Hyperfire Infrared Camera (programmed to 40 cm short-range focus and set to high sensitivity; Reconyx, Holmen, WI, USA) to a stake with a crossbar 50 cm above each tray. While the placement of these 10 × 15 cm cameras above the artificial patches might have reduced the perceived predation risk of small mammals, it was unlikely to be significant as the total cover provided by these cameras 50 cm above the patches was minimal. Moreover, by focusing a camera above the patch and placing a ruler on each tray, we ensured species identification^63^ that would not have been possible with a side mounted camera.

We set cameras to take a series of three photos in a row (1 second apart) and then pause for 1-minute before the camera was triggered again. At each foraging patch we recorded the number of three-picture series taken every 24 hours and the maximum number of each species within each series. To generate a community level metric of activity (*Minute-Visit*) we summed the maximum number of rodents detected in each series. To generate species-specific indices (*Minute-Visit*) we summed the maximum number of individuals of each species in the series. Some foraging patches were visited by avian species. We used the rodents’ percentage of total *Minute-Visits* to estimate the amount of food that rodents consumed, eliminating the impacts of avian consumers. We assumed that the correlation between *Minute-Visits* and *Food Eaten* was the same for both rodents and birds, because it was not possible to approximate the correlation for each group separately due to the unknown rate of food depletion at the patches over each 24-hour period. All applicable institutional and/or national guidelines for the care and use of animals were followed and the project was approved by the University of Florida IAUCC (protocol 201609284).

### Vegetation assessment

At each foraging patch along the gradient, we measured the height of the shrubs and the grass (*Max Ht*). We also included a binary categorical variable (*Binary Ht)* to indicate long grass (>40 cm) or short grass (<40 cm), because savanna rodents appear to respond to grass height at this specific height^26^. In an effort to avoid collinearity we did not use (*Max Ht*) and (*Binary Ht)* in the same models. We estimated the horizontal visual obstruction (*HVO*) of the vegetation surrounding each tray using a Robel pole, averaging the horizontal coverage from measurements taken from 4 m away in each cardinal direction^64^. To quantify fine-scale vegetative cover we estimated the percentage of ground covered (*Ground Cov*) as the combined coverage of shrubs, grass, and forbs looking down onto a 1 m² circular plot from 1.5 m. We estimated the percentage of cover in each quadrant of the circle and then averaged these estimates into a total percentage. We used *HVO* to evaluate cover from visual-based terrestrial predators and *Ground Cov* to quantify cover from avian predators^42^. Finally, to control for the effect of using shrub clusters, we included a binary categorical variable representing *Shrub Size* (Small = 2–4 m³; Large >4 m³).

### Data analysis

To aid in interpretation of our analysis we standardized the directionality of our response variables. To do this, we used the inverse of the GUD, the amount of food eaten (*Food Eaten*) and *Minute-Visit*, both of which decrease with increased perception of risk. To understand rodent communities’ perceptions of fear within and beyond shrubs, we first combined all species’ responses together and evaluated their models to explain variation in the foraging patches. We compared sets of six shrub proximity models (see below) for the response variables *Food Eaten* and *Minute-Visit*. This allowed us to understand if rodents had punctuated responses to shrub cover that fell into broad categories (i.e. shrub vs. grassy areas), or if they were continually increasing, decreasing, or varied at each location. We compared models with distance from the shrub as a continuous variable, a categorical variable for each foraging patch (5 levels), two binary categorizations (*Shrub/Edge* vs. *Grass* and *Shrub* vs. *Edge/Grass*), and three vegetation categories (*Shrub* vs. *Edge* vs. *Grass*). We evaluated each model with generalized linear mixed models, with transect as a random effect, and our count data fitted to negative binomial distributions with a log link function in the package glmmTMB^65^ in Program R (R Version 3.3.3, www.r-project.org, accessed 19 Sep 2017).

Further, we investigated the response (*Food Eaten* and *Minute-Visit*) of all rodents combined to different measures of vegetation structure in grassy areas (i.e *Shrub* and *Edge* excluded). We developed a suite of 15 *a priori* grassy areas models with single variables and additive models using the variables *Max Ht*, *Binary Ht*, *Shrub Size*, *HVO*, and *Ground Cov*. We also modeled a squared term for the *HVO* measurement (*HVO²*) as horizontal visual obstruction may cease benefiting rodents at elevated vegetation densities^66^. We created a model with both *Ground Cov* and *HVO*, as both of these metrics may influence prey perceptions of risk^42^. We also built models with *Max Ht* added to *Ground Cov* and *HVO*, as grass height may also influence rodent foraging and risk perception^28,40^. We standardized (z-score) the vegetation covariates so that their means fell at zero.

We also evaluated the suites of six shrub proximity models and 15 grassy areas models for the activity levels (*Minute-Visit*) of each species individually. For each species we only included activity data from transects where it was detected at least once. We included data from all foraging patches at each of these transects, even if species were not detected at all foraging trays, because we assumed that all foraging patches were available to all foragers. We analyzed these data using negative binomial distributions in glmmTMB^65^ in Program R.

To evaluate all of our suites of models, we ranked them based on their Akaike Information Criterion adjusted for small sample size (AICc^67^), prioritizing the most parsimonious models. We considered models that were <2 AICc units of the best model to be competing models^68^. We evaluated the parameters in these competing models, considering model parameters with β estimates and 95% Confidence Intervals (CI) that did not include 0 to be relevant predictors. For categorical data, we examined the 95% CIs of each category for overlap between them and considered overlapping categories to be redundant. Additionally, we only considered quadratic terms to be important predictors if the 95% CIs of both parameters did not include 0.

## Results

From the small mammal community we detected 7 species of rodent foraging at the foraging trays, of which we had sufficient visits to model 5 of them (Online Resource 4). Omnivorous species were detected at a majority of the 15 transects (*Lemniscomys rosalia*: detected on 14 transects, *Mus minutoides*: 11, *Mastomys natalensis*: 8) while other species were less common on the transects (*Dendromus mystacalis*: detected on 5 transects*, Steatomys pratensis*: 3*, Saccostomus campestris*: 2*, Aethomys ineptus*: 1). Almost all transects (14 out of 15) had at least two species of rodents visiting the foraging patches over the course of 5–7 nights.

### Shrub proximity

**Figure 2:**
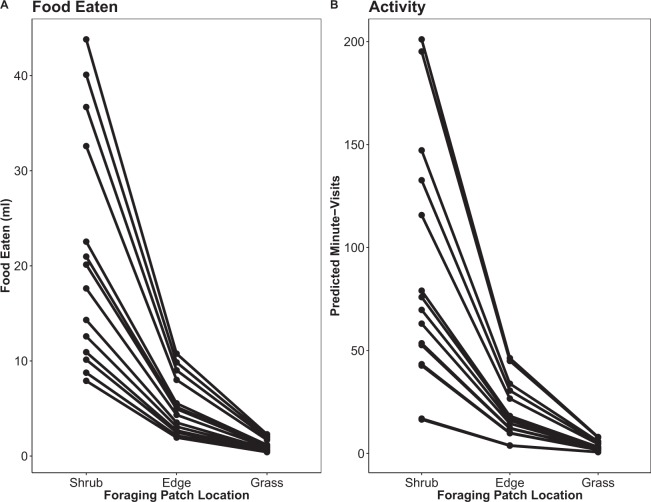
The predicted amount of food eaten (**A**) and activity (**B**) measured as minutes spent at grassy area foraging patches per 24-hour period for all rodents (regardless of species) at each foraging patch in Mbuluzi Game Reserve, Eswatini, from June–August 2016. Predictions were based on the best models that included Shrub, Edge, and Grass as discrete categories (Table 1). Each line represents one transect (n = 15).

The best model, and only competing model, to explain differences in both community *Food Eaten* and community *Minute-Visits* was the model that separated the foraging patches into three categories (*Shrub, Edge, Grass*) where the three grassy area foraging patches were statistically similar (Table 1). The 95% CI of the categories did not overlap zero and there were no other competing models. The model showed that the predicted amount of food that rodents removed from foraging patches was higher under shrub cover than on the edge or in the grassy foraging patches (Fig. 2; *Shrub*: 20.89 ml [16.49, 26.53]; *Edge*: 5.13 ml [3.89, 6.78]; *Grass*: 1.09 ml [0.81, 1.46]). Similarly, community *Minute-Visits* were highest under shrub cover, decreasing at the edge, and lowest at all grass foraging patches (Fig. 2; *Shrub*: 87.01 min. [69.60, 109.17]; *Edge*: 20.03 min. [15.31, 26.28]; *Grass*: 3.46 min. [2.58, 4.64]). We found no indication that the foraging patches away from the shrubs were different from each other or that there was a gradient of response with distance away from the shrub (Table 1).

**Table 1:** Model name, log-likelihood (LL), ΔAICc, model weight (Wt), and parameter, β estimate, standard error (SE), and 95% CI of variables of best competing models (<2 ΔAICc) explaining the amount of food eaten and activity measured as minutes spent at foraging patches per 24-hour period for all rodent foragers.

Model Name^a^	LL	ΔAICc	Wt	Parameter	β	SE	95% CIs
**Food eaten**							
Shrub, Edge, Grass	−740.2	0.0	0.79	Edge*	−1.68	0.35	−2.37–−1.00
				Grass*	−3.51	0.31	−4.13–−2.90
Individual Patch	−739.4	2.7	0.21				
Shrub/Edge, Grass	−749.3	16.3	0.00				
Shrub, Edge/Grass	−753.9	25.5	0.00				
Distance (0–4 m)	−757.6	32.8	0.00				
Null	−775.9	67.4	0.00				
**Activity**							
Shrub, Edge, Grass	−979.9	0.0	0.78	Edge*	−1.75	0.37	−2.47–−1.02
				Grass*	−3.57	0.32	−4.19–−2.95
Individual Patch	−979.1	2.5	0.22				
Shrub, Edge/Grass	−989.8	16.5	0.00				
Shrub/Edge, Grass	−992.4	23.1	0.00				
Distance (0–4 m)	−995.86	29.8	0.00				
Null	−1014.9	65.9	0.00				

Research in Mbuluzi Game Reserve, Eswatini, June–August 2016. Starred responses (*) indicate β estimates of categories with 95% CI outside of zero. Shrub category was set as the reference category. ^a^Shrub, Edge, Grass = 3 foraging patch categories (under shrub, at edge, in grassy area).

Individual Patch = 5 foraging patch categories.

Shrub/Edge, Grass = 2 foraging patch categories, grouping Shrub and Edge together.

Shrub, Edge/Grass = 2 foraging patch categories, grouping Edge and Grass together.

Distance (0–4 m) = gradient of distance from Shrub (0 m) to 1 m, 1.5 m, 2 m, and 4 m into the grass.

### Grassy areas

**Figure 3:**
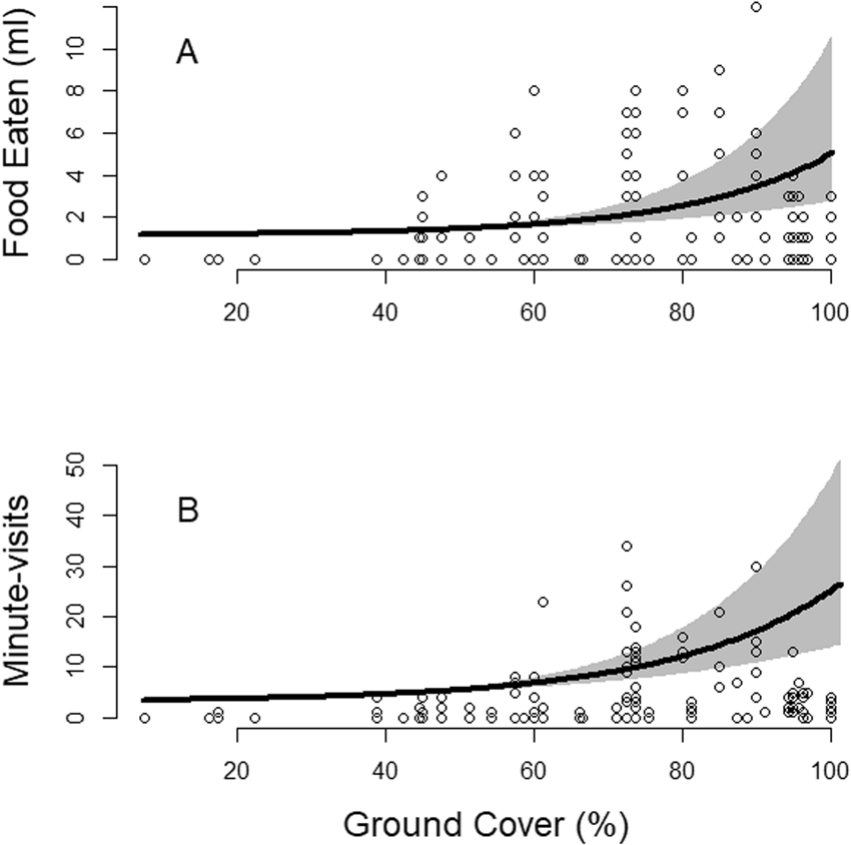
The amount of food eaten (ml) (**A**) and activity (**B**) measured as minutes spent at grassy area foraging patches per 24-hour period for all rodent foragers as a function of percentage ground cover around each foraging patch in Mbuluzi Game Reserve, Eswatini. Black trend line is the result of a generalized linear mixed model fit to a negative binomial distribution and containing ground cover as a relevant variable. Grey area represents the SE.

Examining grassy area foraging patches only, *Food Eaten* and *Minute-Visits* were both best explained by a model with *Ground Cov* (Tables 2, 3). Rodents showed the strongest response to ground cover, with the predicted amount of food eaten and activity increasing from 1 to 5 ml and 3 to 24 minutes respectively (Fig. 3). A curvilinear response of rodents to HVO was also a relevant predictor of *Minute-Visits* (Table 3) with activity peaking around 3.5 decimeters before declining. There were no other relevant variables in competing models (Tables 2, 3).

**Table 2:** Model name, log-likelihood (LL), ΔAICc, model weight (Wt), and parameter, β estimate, standard error (SE), and 95% CI of variables of best competing models (<2 ΔAICc) explaining the amount of food eaten at grassy area foraging patches per 24-hour period for all rodent foragers.

Model Name^a^	LL	ΔAICc	Wt	Parameter	β	SE	95% CIs
Ground Cov + HVO + HVO²	−238.5	0.0	0.28	Ground Cov*	0.89	0.45	0.21–1.57
				HVO*	0.66	0.31	0.05–1.26
				HVO²	−0.33	0.18	−0.68–0.02
Ground Cov	−240.8	0.4	0.23	Ground Cov*	1.28	0.34	0.60–1.96
Ground Cov + HVO	−240.1	1.1	0.16	Ground Cov*	1.18	0.36	0.57–1.86
				HVO	0.30	0.26	−0.20–0.81
Shrub Size + Ground Cov	−240.7	2.1	0.10				
Ground Cov + Max Ht	−240.7	2.2	0.09				
Ground Cov + Max Ht + HVO + HVO²	−240.0	3.0	0.06				
HVO + HVO²	−242.0	4.7	0.03				
Binary Ht	−243.3	5.3	0.02				
Max Ht + HVO + HVO²	−241.8	6.6	0.01				
Shrub + HVO + HVO²	−241.9	6.8	0.01				
HVO + Max Ht	−243.9	8.5	0.00				
Max Ht	−245.1	8.8	0.00				
HVO	−245.6	9.9	0.00				
Shrub Size + Max Ht	−244.8	10.4	0.00				
Null	−247.9	12.4	0.00				

Research in Mbuluzi Game Reserve, Eswatini, June–August 2016. Starred responses (*) indicate β estimates of categories with 95% CI outside of zero.

^a^Ground Cov = combined coverage of shrubs, grass, and forbs looking down onto a 1 m² circular plot from 1.5 m.

HVO = horizontal visual obstruction based on Robel pole (Robel *et al*. 1970).

HVO² = horizontal visual obstruction based on Robel pole (Robel *et al*. 1970), squared.

Max Ht = height of grass (dm).

Shrub Size = 2 categories of shrub size (2–3 m³ and >4 m³).

Binary Ht = 2 categories of grass height (>40 cm and <40 cm).

**Table 3:** Model name, log-likelihood (LL), ΔAICc, model weight (Wt), and parameter, β estimate, standard error (SE), and 95% CI of variables of best competing models (<2 ΔAICc) explaining the activity measured as minutes spent at foraging patches per 24-hour period for all rodent foragers.

Model Name^a^	ΔAICc	LL	Wt	Parameter	β	SE	95% CIs
Ground Cov + HVO + HVO²	0.0	−345.5	0.42	Ground Cov*	0.78	0.30	0.20–1.37
				HVO*	0.63	0.27	0.10–1.17
				HVO²*	−0.33	0.15	−0.62–−0.04
Ground Cov	1.7	−348.5	0.18	Ground Cov*	1.12	0.29	0.55–1.70
Ground Cov + HVO	2.6	−347.9	0.12				
Ground Cov + Max Ht	3.3	−348.2	0.08				
Shrub Size + Ground Cov	3.3	−348.2	0.08				
Ground Cov + Max Ht + HVO + HVO²	4.4	−347.7	0.05				
HVO + HVO²	5.0	−349.1	0.04				
Max Ht + HVO + HVO²	6.9	−349.0	0.01				
Shrub + HVO + HVO²	7.1	−349.1	0.01				
HVO + Max Ht	10.6	−351.9	0.00				
Max Ht	10.8	−353.0	0.00				
Binary Ht	11.9	−353.6	0.00				
HVO	12.1	−353.7	0.00				
Shrub Size + Max Ht	12.5	−352.8	0.00				
Null	14.9	−356.1	0.00				

Research in Mbuluzi Game Reserve, Eswatini, June–August 2016. Starred responses (*) indicate β estimates of categories with 95% CI outside of zero. Shrub category was set as the reference category.

^a^Ground Cov = combined coverage of shrubs, grass, and forbs looking down onto a 1 m² circular plot from 1.5 m.

HVO = horizontal visual obstruction based on Robel pole (Robel *et al*. 1970).

HVO² = horizontal visual obstruction based on Robel pole (Robel *et al*. 1970), squared.

Max Ht = height of grass (dm).

Shrub Size = 2 categories of shrub size (2–3 m³ and >4 m³).

Binary Ht = 2 categories of grass height (>40 cm and <40 cm).

### Individual species

**Figure 4:**
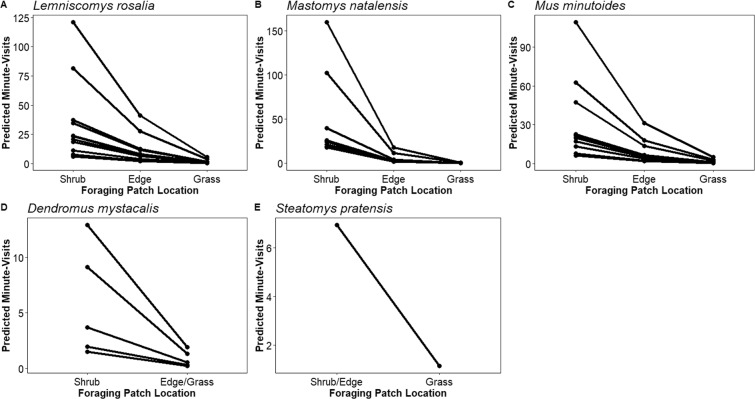
The predicted activity measured as minutes spent at foraging patches per 24-hour period for the most commonly-detected rodent species. Research in Mbuluzi Game Reserve, Eswatini, June–August 2016. Individual lines represent the predictions based on competing models for each species (Online Resource 5).

Species responses to shrub proximity were similar to the community response, with individuals spending more time at *Shrub* foraging patches than *Edge* foraging patches and rarely visiting *Grass* foraging patches (Fig. 4; Online Resource 5). The visiting patterns of *L. rosalia* and *M. minutoides* were best described by a model with three categories (*Shrub, Edge, Grass*) and no competing models. The best models explaining the response of *M. natalensis* included a model with each individual patch, a model with distance between patches, and a model with three categories (*Shrub, Edge, Grass*). Finding considerable overlap between the 95% CIs of categories estimates in the individual patch model, we considered the three categories and distance models to be better representations of the data. The best models for explaining the visits of *D. mystacalis* and *S. pratensis* had two categories. The two-category model best explaining *D. mystacalis* activity was *Shrub* vs. *Edge/Grass*, while the combined category for *S. pratensis* was *Shrub/Edge vs. Grass*. Adding more categories to these models did not improve parsimony.

Each species responded differently to variation in vegetation structure in the grassy areas. Examining the relevant parameters in competing models (Online Resource 6), one species, *L. rosalia*, consistently increased *Minute-Visits* with increasing ground cover (β 1.86 [1.11–2.60]). One species, *M. minutoides*, showed a curvilinear relationship with *HVO* (*HVO* = β 6.00 [2.50–9.51], *HVO*^2^ = β −5.25 [−8.46–1.85]), increasing its visits up to 30 cm before declining. Two species appeared to responded to *Max Ht* with *S. pratensis* showing a significant increase in visits with grass height (β 1.88 [0.04–3.73]) and *M. natalensis* potentially decreasing visits with grass height (β −1.13 [−2.27–0.00]).

## Discussion

The rodent species in this study showed sharp declines in their perceived predation risks underneath and on the edge of shrub cover. This perception did not slowly increase with distance from the shrub, rather it was punctuated. The perception of risk increased at the edge of the shrub and again in open areas only 50 cm away from the edge. Despite considerable variation in the body mass, ecology, activity periods (i.e. nocturnal and crepuscular), and habitat use of the rodents in this study^57^, their perception of risk with regards to shrubs was consistent. Variation in species responses, although subtle, was more pronounced in grassy areas. Our findings suggest that the shrub encroachment of >40% coverage (doubling after 10 years^52^) found on our site^12^ and throughout many African savannas^3^ may be altering and homogenizing rodents’ perceptions of predation risk and shaping their use of space^27,33^.

The rodents’ consistent response to shrub cover allows us to consider the community-wide responses to anthropogenically altered savannas. When woody cover has been drastically reduced through firewood harvesting or extensive browsing, we would expect sharp increases in rodents’ perceived risks of predation^69^. This enhanced fear may help explain the pattern of depauperate rodent communities in savannas with reduced cover, but where ample food resources persist^12,22,44^. These communities are often dominated by one generalist species such as *Mastomys natalensis*^36^. In our study, *Mastomys natalensis* increased activity with decreasing grass height and has been shown to increase activity in open and disturbed environments where food is plentiful^70^.

We would expect most rodent species to thrive in the relatively safe environment of thick shrub cover^22,36,44^ presumably created by altered disturbance regimes and increasing atmospheric CO_2_. However, there is clearly a threshold where the benefits of safety from shrub cover are outweighed by the loss of foraging opportunities. Most savanna rodents rely heavily on grass or grass seeds for food^61^ and increasing shrubs can reduce the amount of grass and forage for herbivores^13,71^. This might explain the pattern common in other studies where rodent diversity increased with increasing shrub cover until a threshold was reached where diversity declined^12,38^.

The consistent and predictable response of rodents also suggests that woody vegetation, by altering animals’ perception of fear, may act as an ecological filter, selecting against certain species or traits such as body size^72^. The reduced activity and diversity of large mammals in savannas with extensive shrub cover cannot be explained by reductions in food alone^16^ and increasingly appears to be a function of perceived predation risk^18–20,34^ from reduced sight lines and escape routes. Alternatively, small-bodied rodents appear to increase activity and diversity around shrubs^12^, potentially due to reductions in their perception of risk^36^, as demonstrated in our study. Additionally, the known reduction of large mammals from areas with extensive shrub cover might enhance rodent populations because they are released from competition for resources^73^. Overall, there is a clear pattern where the perception of risk from woody vegetation favors smaller mammalian herbivores over larger ones. The potential consequence of this is a trophic shift, or a replacement of large consumers with smaller ones, which is likely to have a marked influence on plant community dynamics and composition^73–75^. The loss and replacement of large animals is a global conservation pattern, but rarely has it been associated with a specific mechanism^73–75^.

Away from shrubs in grassy areas, the rodents’ perceived risk of predation changed with ground cover, although metrics of safety (*Minute-Visits*, *Food Eaten*) were <20% of those under shrubs. We expected that reductions in ground cover, grass height and horizontal structure would increase rodents’ perception of risks^28,39,40^. While perceived predation risk for *Steatomys pratensis* increased with a reduction in grass height, *Mastomys natalensis* showed the reverse relationship. *Mus minutoides* showed a heightened perception of risk with reduced horizontal cover, though its response was curvilinear, with increased perceptions of risk with extensive horizontal cover as well. In general, species’ responses to vegetation varied with habitat structure once they were away from shrubs. Accordingly, the heterogeneity of structure in areas away from shrubs will likely lead to variation in different species’ perceptions of fear, potentially fostering species coexistence and diversity^25^. However, the influence of structure in grassy areas on rodents’ perception of fear was minimal when compared to the influence of shrub cover.

In our study, the patterns of rodent behavior appear consistent with broader patterns of rodent communities in African savannas. However, there are likely a host of other processes shaping rodents’ behaviors and community dynamics. For example, we were not able to separate out the influence of rodent species interactions from predation risk, with aggression and attraction potentially influencing variation in species foraging and activity^76^. Nevertheless, because we saw consistency between community and species-level responses, it appears that the influence of species interactions on our response metrics was minimal. Additionally, there is considerable evidence that rodents can be influenced by the broader patch and landscape level variation in woody cover^77^. At these scales, woody cover may alter rodent movement patterns^78^, food resources^13,71^, predator communities^79^, and overall perception of risk^77^. Replicating this work on sites with varying levels of broad-scale encroachment might allow us to parse the relative influence of the broader vs. fine scale vegetation structure on rodents’ behaviors and community dynamics.

Due to its consistent and powerful influence on rodent fine-scale perceptions of fear, shrub cover is likely to have a strong influence on the species and communities that rely on it for safety. In turn, the greatly reduced perceived risk of predation in and around shrubs might provide a mechanistic explanation for the patterns of reduced rodent diversity seen in open savannas^36,80,81^. Species’ perceptions of fear can have a strong influence on their reproductive fitness, foraging, movements, and physiology^69,82–84^. With shrubs clearly shaping rodents’ perception of fear, there can be little doubt that the anthropogenic forces shaping woody vegetation will have a powerful influence on the rodent species and communities in African savannas.

## Supplementary information

Supplementary materials

## References

[1] Andersen AN, Woinarski JCZ, Parr CL (2012). Savanna burning for biodiversity: fire management for faunal conservation in Australian tropical savannas. Austral Ecol..

[2] Parr CL, Lehmann CER, Bond WJ, Hoffman WA, Andersen AN (2014). Tropical grassy biomes: misunderstood, neglected, and under threat. Trends Ecol Evol..

[3] Stevens N, Lehmann CER, Murphy BP, Durigan G (2017). Savanna woody encroachment is widespread across three continents. Global Change Biol.

[4] Roques KG, O’Connor TG, Watkinson AR (2001). Dynamics of shrub encroachment in an African savanna: relative influences of fire, herbivory, rainfall and density dependence. J Appl Ecol..

[5] Wigley BJ, Bond WJ, Hoffman MT (2010). Thicket expansion in a South African savanna under divergent land use: local vs. global drivers?. Global Change Biol..

[6] Stanton Richard A., Boone Wesley W., Soto-Shoender Jose, Fletcher Robert J., Blaum Niels, McCleery Robert A. (2017). Shrub encroachment and vertebrate diversity: A global meta-analysis. Global Ecology and Biogeography.

[7] Levick SR, Asner GP, Kennedy-Bowdoin T, Knapp DE (2009). The relative influence of fire and herbivory on savanna three-dimensional vegetation structure. Biol Conserv..

[8] Hejcmanova P, Hejcman M, Camara AA, Antoninova M (2010). Exclusion of livestock grazing and wood collection in dryland savannah: an effect on long-term vegetation succession. Afr J Ecol.

[9] Foster CN, Barton PS, Lindenmayer DB (2014). Effects of large native herbivores on other animals. J Appl Ecol.

[10] Mograbi PJ (2015). Biomass Increases Go under Cover: Woody Vegetation Dynamics in South African Rangelands. PLoS ONE.

[11] Ogada DL (2008). Impacts of large herbivorous mammals on bird diversity and abundance in an African savanna. Oecologia.

[12] McCleery RA (2018). Animal diversity declines with broad-scale homogenization of canopy cover in African savannas. Biol Conserv.

[13] Soto-Shoender JR, McCleery RA, Monadjem A, Gwinn DC (2018). The importance of grass cover for mammalian diversity and habitat associations in a bush encroached savanna. Biol Conserv.

[14] Archer SR, Predick KI (2014). An ecosystem services perspective on brush management: research priorities for competing land-use objectives. J Ecol.

[15] Eldridge DJ (2011). Impacts of shrub encroachment on ecosystem structure and functioning: towards a global synthesis. Ecol lett.

[16] Stuart‐Hill GC, Tainton NM (1989). The competitive interactions between Acacia karroo and the herbaceous layer and how this is influenced by defoliation. J Appl Ecol.

[17] Mugasi SK, Sabiiti EN, Tayebwa BM (2000). The economic implications of bush encroachment on livestock farming in rangelands of Uganda. Afr J of Range For Sci.

[18] Ford AT (2014). Large carnivores make savanna tree communities less thorny. Science.

[19] Riginos C (2015). Climate and the landscape of fear in an African savanna. J Anim Ecol.

[20] LeRoux E, Kerley GIH, Cromsigt JPGM (2018). Megaherbivores modify trophic cascades triggered by fear of predation in an African savanna ecosystem. Curr Biol.

[21] Mohr K (2003). Foraging of multimammate mice, Mastomys natalensis, under different predation pressure: cover, patch-dependent decisions and density-dependent GUDs. Oikos.

[22] Hagenah N, Prins HHT, Olff H (2009). Effects of Large Herbivores on Murid Rodents in a South African Savanna. J Trop Ecol.

[23] Wheeler HC, Hik DS (2014). Giving-up densities and foraging behaviour indicate possible effects of shrub encroachment on arctic ground squirrels. Anim Behav.

[24] Verdolin JL (2006). Meta-analysis of foraging and predation risk trade-offs in terrestrial systems. Behav Ecol Sociobiol.

[25] Kotler BP (1984). Risk of Predation and the Structure of Desert Rodent Communities. Ecology.

[26] Kotler BP, Brown JS, Hasson O (1991). Factors Affecting Gerbil Foraging Behavior and Rates of Owl Predation. Ecology.

[27] Abu Baker MA, Brown JS (2014). Foraging in space and time structure an African small mammal community. Oecologia.

[28] Banasiak N, Shrader AM (2015). Similarities in perceived predation risk prevent temporal partitioning of food by rodents in an African grassland. J Mammal.

[29] Shrader AM, Brown JS, Kerley GIH, Kotler BP (2008). Do free-ranging domestic goats show “landscapes of fear”? Patch use in response to habitat features and predator cues. J Arid Environ.

[30] Heithaus MR, Wirsing AJ, Burkholder D, Thomson J, Dill LM (2009). Towards a predictive framework for predator risk effects: the interaction of landscape features and prey escape tactics. J Anim Ecol.

[31] Lima SL, Dill LM (1990). Behavioral decisions made under the risk of predation: a review and prospectus. Can J of Zoo.

[32] Schmitz OJ, Beckerman AP, O’Brien KM (1997). Behaviorally Mediated Trophic Cascades: Effects of Predation Risk On Food Web Interactions. Ecology.

[33] Laundré JW, Hernandez L, Ripple WJ (2010). The Landscape of Fear: Ecological Implications of Being Afraid. The Open Ecol J.

[34] Underwood R (1982). Vigilance Behaviour in Grazing African Antelopes. Behaviour.

[35] Tadesse SA, Kotler BP (2013). The impacts of humans and livestock encroachments on the habitats of mountain nyala (Tragelaphus buxtoni) in Munessa, Ethiopia. Int J Biodiv Conserv.

[36] Loggins AA (2019). Vegetation structure shapes small mammal communities in African savannas. J Mammal.

[37] Avenant N (2011). The potential utility of rodents and other small mammals as indicators of ecosystem ‘integrity’ of South African grasslands. Wildl Res.

[38] Bergstrom BJ, Sensenig RL, Augustine DJ, Young TP (2018). Searching for cover: soil enrichment and herbivore exclusion, not fire, enhance African savanna small-mammal abundance. Ecosphere.

[39] Long AK (2012). Multi-scale habitat selection of Mus minutoides in the Lowveld of Swaziland. Afr J Ecol.

[40] Jacob J, Brown JS (2000). Microhabitat use, giving-up densities and temporal activity as short-and long-term anti-predator behaviors in common voles. Oikos.

[41] Kotler BP, Brown JS, Hasson O (1991). Factors affecting gerbil foraging behavior and rates of owl predation. Ecology.

[42] Embar K, Kotler BP, Mukherjee S (2011). Risk management in optimal foragers: the effect of sightlines and predator type on patch use, time allocation, and vigilance in gerbils. Oikos.

[43] Morris DW, Davidson DL (2000). Optimally foraging mice match patch use with habitat differences in fitness. Ecology.

[44] Kerley GI (1992). Ecological correlates of small mammal community structure in the semi‐arid Karoo, South Africa. J Zool.

[45] Wiens JA (1989). Spatial scaling in ecology. Funct Ecol.

[46] Charnov EL (1976). Optimal Foraging, the Marginal Value Theorem. Theor Popul Biol.

[47] Brown JS (1988). Patch Use as an Indicator of Habitat Preference, Predation Risk, and Competition. Behav Ecol Sociobiol.

[48] Brown JS, Laundre JW, Gurung M (1999). The Ecology of Fear: Optimal Foraging, Game Theory, and Trophic Interactions. J Mammal.

[49] Matondo JI, Peter G, Msibi KM (2004). Evaluation of the impact of climate change on hydrology and water resources in Swaziland: Part 1. Phys Chem Earth Pt A/B/C.

[50] Matondo JI, Peter G, Msibi KM (2005). Managing water under climate change for peace and prosperity in Swaziland. Phys Chem Earth Pt A/B/C.

[51] Roques KG, O’connor TG, Watkinson AR (2001). Dynamics of shrub encroachment in an African savanna: relative influences of fire, herbivory, rainfall and density dependence. J App Ecol.

[52] Sirami C, Monadjem A (2012). Changes in bird communities in Swaziland savannas between 1998 and 2008 owing to shrub encroachment. Divers and Distrib.

[53] Monadjem, A. Mammals of Swaziland. (Conservation Trust of Swaziland, 1998).

[54] Blanc J. J. *et al*. African elephant status report 2002: An update from the African Elephant Database (Occasional Paper of the IUCN Species Survival Commission, No. 29, 2003).

[55] Monadjem A (1997). Habitat preferences and biomasses of small mammals in Swaziland. Afr J Ecol.

[56] Monadjem A (1999). Population dynamics of Mus minutoides and Steatomys pratensis (Muridae: Rodentia) in a subtropical grassland in Swaziland. Afr J Ecol.

[57] Skinner, J. D. & Chimimba, C. T. *The Mammals of the Southern African Subregion*. (Cambridge University Press, 2005)

[58] Hurst ZM (2013). Dynamic edge effects in small mammal communities across a conservation-agricultural interface in Swaziland. PLoS ONE.

[59] Monadjem A (1997). Stomach contents of 19 species of small mammals from Swaziland. S Afr J Zool.

[60] Bergstrom BJ (2013). Would East African savanna rodents inhibit woody encroachment? Evidence from stable isotopes and microhistological analysis of feces. J Mammal.

[61] Monadjem A, Perrin MR (2003). Population fluctuations and community structure of small mammals in a Swaziland grassland over a three-year period. Afr Zool.

[62] Bedoya-Perez MA (2013). A practical guide to avoid giving up on giving-up densities. Behav Ecol Sociobiol.

[63] McCleery RM (2014). A Novel Method for Camera-Trapping Small Mammals. Wildlife Soc B.

[64] Robel RJ, Briggs JN, Dayton AD, Hulbert LC (1970). Relationships Between Visual Obstruction Measurements and Weight of Grassland Vegetation. J Range Manage.

[65] Brooks ME (2017). glmmTMB Balances Speed and Flexibility Among Packages for Zero-inflated Generalized Linear Mixed Modeling. The R Journal.

[66] Kotler BP, Blaustein L, Brown JS (1992). Predator facilitation: the combined effect of snakes and owls on the foraging behavior of gerbils. Ann Zool Fennici.

[67] Akaike, H. Information theory as an extension of the maximum likelihood principle in 2nd International Symposium on Information Theory (eds Petrov, B. N. & Csaksi, F.) 267–281 (Akademiai Kiado, 1973).

[68] Burnham K. P. & Anderson, D. R. (1998) *Model Selection and Inference* (Springer, 1988).

[69] Preisser EL, Bolnick DI, Benard MF (2005). Scared to death? The effects of intimidation and consumption in predator-prey interactions. Ecology.

[70] Monadjem A, Perrin MR (1998). The effect of supplementary food on the home range of the multimammate mouse, Mastomys natalensis. S. Afr J Ecol.

[71] Smit IPJ, Prins HHT (2015). Predicting the effects of woody encroachment on mammal communities, grazing biomass and fire frequency in African savannas. PLoS ONE.

[72] Duflot R (2014). Landscape heterogeneity as an ecological filter of species traits. Acta Oecol.

[73] Keesing F, Young TP (2014). Cascading consequences of the loss of large mammals in an African savanna. Bioscience.

[74] Estes JA (2011). Trophic downgrading of planet earth. Science.

[75] Malhi Y (2016). Megafauna and ecosystem function from the Pleistocene to the Anthropocene. P Natl Acad Sci USA.

[76] Brown JS (1999). Vigilance, patch use and habitat selection: foraging under predation risk. Evol Ecol Res.

[77] Laundré JW (2014). The landscape of fear: the missing link to understand top-down and bottom-up controls of prey abundance?. Ecology.

[78] Wegner JF, Merriam G (1979). Movements by birds and small mammals between a wood and adjoining farmland habitats. J Appl Ecol.

[79] Blaum N, Rossmanith E, Popp A, Jeltsch F (2007). Shrub encroachment affects mammalian carnivore abundance and species richness in semiarid rangelands. Acta Oecologica.

[80] van Deventer M, Nel JAJ (2006). Habitat, food, and small mammal community structure in Namaqualand. Koedoe.

[81] Lagesse JV, Thondhlana G (2016). The effect of land-use on small mammal diversity inside and outside the Great Fish River Nature Reserve, Eastern Cape, South Africa. J Arid Environ.

[82] Orrock JL, Danielson BJ, Brinkerhoff RJ (2004). Rodent foraging is affected by indirect, but not by direct, cues of predation risk. Behav Ecol.

[83] Zanette LY, White AF, Allen MC, Clinchy M (2011). Perceived predation risk reduces the number of offspring songbirds produce per year. Science.

[84] Clinchy M, Sheriff MJ, Zanette LY (2013). Predator-induced stress and the ecology of fear. Funct Ecol.

