# Supplementary material for: Shrub cover homogenizes small mammals’ activity and perceived predation risk
Source: Sci Rep. 2019 Nov 14;9:16857. doi: 10.1038/s41598-019-53071-y (PMC6856081; doi:10.1038/s41598-019-53071-y)
Supplement: Supplementary file 1 — Supplementary materials [file 41598_2019_53071_MOESM1_ESM.docx]

**SUPPLEMENTARY INFORMATION**

**Shrub cover homogenizes small mammals’ activity and perceived predation risk**

Anne A. Loggins^1,4^, Adrian M. Shrader^2,^ Ara Monadjem^3,2^, Robert A. McCleery^2, 4^

^1^*School of Natural Resources and the Environment, University of Florida, Gainesville, Florida, USA*

^2^ *Mammal Research Institute, Department of Zoology & Entomology, University of Pretoria, Private Bag 20, Hatfield 0028, Pretoria, South Africa*

^3^*Department of Biological Sciences, University of Eswatini, Private Bag 4, Kwaluseni, Eswatini*

^4^*Departemen*t of *Wildlife Ecology and Conservation, University of Florida, Gainesville, Florida, USA*

[aloggins@alumni.stanford.edu](mailto:aloggins@alumni.stanford.edu), +1 (206) 745-0165 (*AAL*)

[adrian.shrader@up.ac.za](mailto:adrian.shrader@up.ac.za), +27 (0)12 420-3232 *(AMS)*

[ara@uniswa.sz](mailto:aramsotoonadjem@gmail.com), +268 2517 0378 (*AM*)

[ramccleery@ufl.edu](mailto:ramccleery@ufl.edu), +1 (352) 846-0566 (*RAM*); Corresponding author


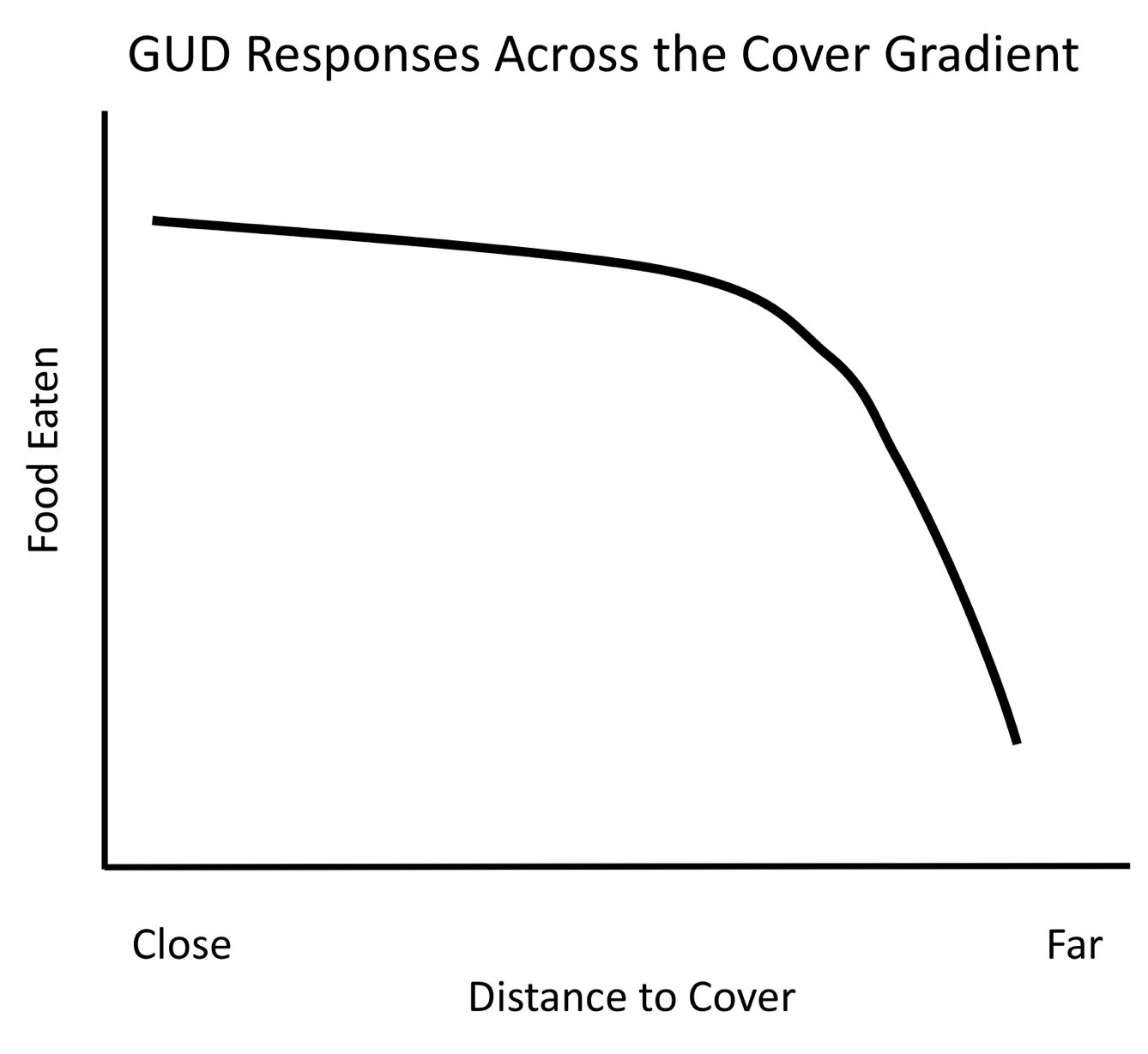


Online Resource 1. Predicted outcomes of giving up density (GUD) experiments along a shrub cover gradient from near to far from cover.

Online Resource 2. Habitat preferences, average adult mass (g), and diet (Skinner and Chimimba 2005) of small mammal species detected in foraging patches in Mbuluzi Game Reserve, Eswatini, from June-August 2016.

| Species | Mass | Diet | Habitat |
| --- | --- | --- | --- |
| *Aethomys ineptus* | 78 | Granivore-herbivore | Grassland and woodland |
| *Dendromys mystacalis* | 8 | Granivore-insectivore | Grassland with shrubs, tall grass |
| *Gerbilliscus leucogaster* | 70 | Omnivore | Savanna and woodland, burrows |
| *Lemniscomys rosalia* | 57 | Herbivore-granivore | Grassland, tall grass |
| *Mastomys natalensis* | 46 | Granivore-omnivore | Wide tolerance, savanna, agriculture |
| *Mus minutoides* | 6 | Omnivore | Wide tolerance, savanna, agriculture |
| *Saccostomus campestris* | 48 | Granivore | Savanna and woodland, burrows |
| *Steatomys pratensis* | 23 | Graminivore- granivore | Open grassland and woodland, burrows |


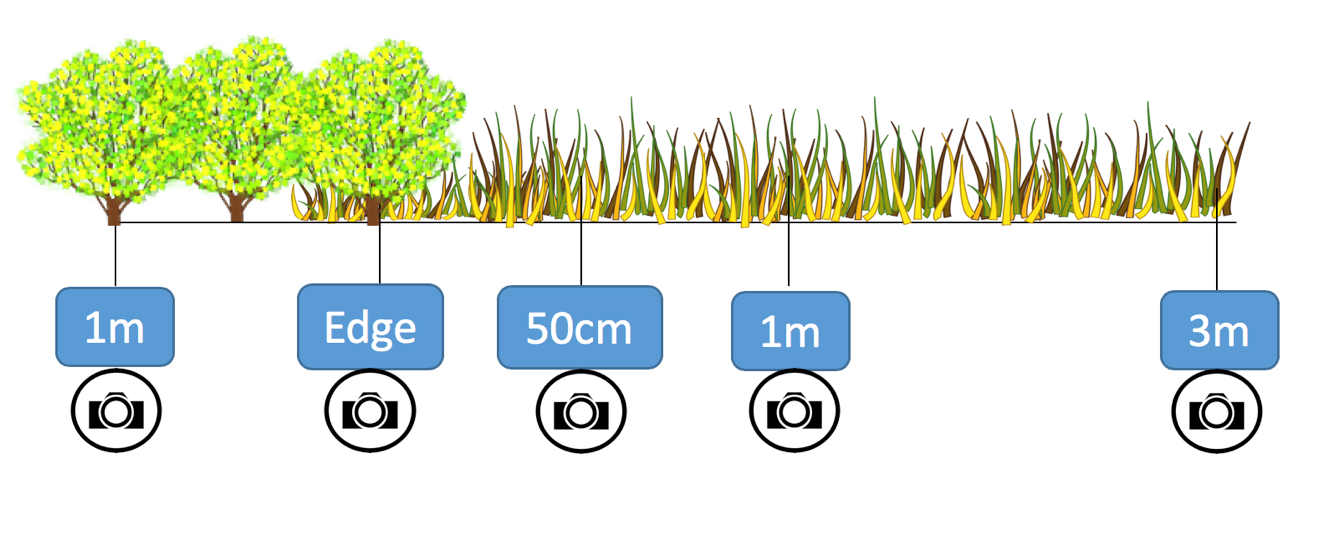


Online Resource 3. A depiction of the locations of the foraging patches with camera traps 1 m within a shrub, at the edge between shrub and grassland, and 50 cm, 1 m, and 3 m away from the shrub into open grassy area in Mbuluzi Game Reserve, Eswatini.

Online Resource 4. The number of nights each species of rodent was detected at each foraging patch in Mbuluzi Game Reserve, Eswatini, from June-August 2016.

| Species |  | Shrub | Edge | 50cm | 1m | 3m |
| --- | --- | --- | --- | --- | --- | --- |
| *Lemniscomys rosalia* |  | 67 | 47 | 14 | 16 | 14 |
| *Mus minutoides* |  | 52 | 31 | 10 | 8 | 15 |
| *Mastomys natalensis* |  | 37 | 16 | 6 | 1 | 1 |
| *Dendromus mystacalis* |  | 17 | 6 | 6 | 6 | 4 |
| *Steatomys pratensis* |  | 9 | 6 | 4 | 1 | 4 |
| *Aethomys ineptus* |  | 1 | 0 | 4 | 0 | 0 |
| *Saccostomus campestris* |  | 1 | 0 | 0 | 0 | 1 |

Online Resource 5. Model name, log-likelihood (LL), ΔAICc, model weight (WT), and parameter, β estimate, standard error (SE), and 95% CI of variables of best competing models (< ΔAICc) explaining the activity measured as minutes spent at foraging patches per night for the most commonly detected rodent foragers (*Lemniscomys rosalia*, *Mus minutoides*, *Mastomys natalensis*, *Dendromus mystacalis, Steatomys pratensis*). Research in Mbuluzi Game Reserve, Eswatini, June-August 2016. Starred responses (*) indicate β estimates of categories with 95% CI outside of zero. Shrub category was set as the reference category.

| Model Name^a^ | LL | ΔAICc | Wt | Parameter | β | SE | 95% CIs |
| --- | --- | --- | --- | --- | --- | --- | --- |
| ***Lemniscomys rosalia*** |  |  |  |  |  |  |  |
| Shrub, Edge, Grass | -674.0 | 0.0 | 0.83 | Edge * | -1.24 | 0.43 | -2.08– -0.39 |
|  |  |  |  | Grass * | -3.81 | 0.42 | -4.63– -2.99 |
| Individual Patch | -673.8 | 4.0 | 0.12 |  |  |  |  |
| Shrub, Edge/Grass | -677.6 | 5.3 | 0.06 |  |  |  |  |
| Distance (0-4m) | -686.1 | 22.3 | 0.00 |  |  |  |  |
| Shrub/Edge, Grass | -690.0 | 30.0 | 0.00 |  |  |  |  |
| Null | -704.9 | 57.8 | 0.00 |  |  |  |  |
| ***Mus minutoides*** |  |  |  |  |  |  |  |
| Shrub, Edge, Grass | -471.2 | 0.0 | 0.68 | Edge * | -1.46 | 0.63 | -2.69– -0.23 |
|  |  |  |  | Grass * | -3.82 | 0.57 | -4.96– -2.69 |
| Shrub, Edge/Grass | -473.8 | 2.9 | 0.16 |  |  |  |  |
| Individual Patch | -470.6 | 2.9 | 0.16 |  |  |  |  |
| Shrub, Edge/Grass | -478.9 | 13.2 | 0.00 |  |  |  |  |
| Shrub/Edge, Grass | -480.8 | 17.1 | 0.00 |  |  |  |  |
| Null | -489.5 | 32.4 | 0.00 |  |  |  |  |
| ***Mastomys natalensis*** |  |  |  |  |  |  |  |
| Individual Patch | -279.7 | 0.0 | 0.45 | Edge * | -2.53 | 0.37 | -3.64– -1.43 |
|  |  |  |  | 50cm * | -4.18 | 0.68 | -5.51– -2.84 |
|  |  |  |  | 1m * | -6.29 | 1.16 | -8.57– -4.02 |
|  |  |  |  | 3m * | -6.35 | 1.17 | -8.63– -4.07 |
| Distance (0-4m) | -283.5 | 0.9 | 0.30 | Dist* | -2.28 | 0.64 | -3.15– -1.85 |
| Shrub, Edge, Grass | -282.5 | 1.2 | 0.25 | Edge * | -2.56 | 0.61 | -3.75– -1.36 |
|  |  |  |  | Grass * | -5.27 | 0.63 | -6.50– -4.04 |
| Shrub/Edge, Grass | -289.0 | 11.8 | 0.00 |  |  |  |  |
| Shrub, Edge/Grass | -290.1 | 14.1 | 0.00 |  |  |  |  |
| Null | -305.1 | 42.0 | 0.00 |  |  |  |  |
| ***Dendromus mystacalis*** |  |  |  |  |  |  |  |
| Shrub, Edge/Grass | -147.8 | 0.0 | 0.66 | Grass * | -2.17 | 0.54 | -3.23– -1.11 |
| Shrub, Edge, Grass | -147.7 | 2.1 | 0.23 |  |  |  |  |
| Distance (0-4m) | -150.1 | 4.7 | 0.06 |  |  |  |  |
| Individual Patch | -147.5 | 6.3 | 0.03 |  |  |  |  |
| Shrub, Edge/Grass | -151.3 | 7.1 | 0.02 |  |  |  |  |
| Null | -154.0 | 10.2 | 0.00 |  |  |  |  |
| ***Steatomys pratensis*** |  |  |  |  |  |  |  |
| Shrub/Edge, Grass | -103.7 | 0.0 | 0.29 | Grass * | -1.32 | 0.39 | -2.35– -0.29 |
| Shrub, Edge/Grass | -103.7 | 0.1 | 0.28 | Grass * | -1.39 | 0.41 | -2.30– -0.49 |
| Shrub, Edge, Grass | -102.6 | 0.4 | 0.24 | Edge | -0.85 | 0.51 | -1.85– 0.15 |
|  |  |  |  | Grass * | -1.65 | 0.46 | -2.56– -0.75 |
| Individual Patch | -101.1 | 2.3 | 0.09 |  |  |  |  |
| Distance (0-4m) | -105.2 | 3.0 | 0.06 |  |  |  |  |
| Null | -106.8 | 3.9 | 0.04 |  |  |  |  |

^a^ Shrub, Edge, Grass = 3 foraging patch categories (under shrub, at edge, in grassy area)

Individual Patch = 5 foraging patch categories

Shrub/Edge, Grass = 2 foraging patch categories, grouping Shrub and Edge together

Shrub, Edge/Grass = 2 foraging patch categories, grouping Edge and Grass together

Distance (0-4m) = gradient of distance from Shrub (0m) to 1m, 1.5m, 2m, and 4m into the grass

Online Resource 6. Model name, log-likelihood (LL), ΔAICc, model weight (WT), and parameter, β estimate, standard error (SE), and 95% CI of variables of best competing models (< ΔAICc) explaining the activity measured as minutes spent at grassy area foraging patches per night for the most commonly detected rodent foragers (*Lemniscomys rosalia*, *Mus minutoides*, *Mastomys natalensis*, *Dendromus mystacalis, Steatomys pratensis*). Research in Mbuluzi Game Reserve, Eswatini, June-August 2016. Starred responses (*) indicate β estimates of categories with 95% CI outside of zero.

| Model Name^a^ | LL | ΔAICc | Wt | Parameter | β | SE | 95% CIs |
| --- | --- | --- | --- | --- | --- | --- | --- |
| ***Lemniscomys rosalia*** |  |  |  |  |  |  |  |
| GroundCov+Max Ht | -182.5 | 0.0 | 0.31 | GroundCov* | 1.86 | 0.38 | 1.11–2.60 |
|  |  |  |  | MaxHt | -0.55 | 0.34 | -1.21–0.11 |
| GroundCov | -183.8 | 0.6 | 0.23 | GroundCov* | 1.49 | 0.30 | 0.91–2.08 |
| GroundCov+MaxHt+HVO+ HVO² | -180.7 | 0.8 | 0.21 | GroundCov* | 1.36 | 0.43 | 0.53–2.02 |
|  |  |  |  | MaxHt | -0.77 | 0.35 | -1.45–-0.09 |
|  |  |  |  | HVO | 1.84 | 0.98 | -0.08–3.77 |
|  |  |  |  | HVO²* | -1.57 | 0.87 | -3.27–-0.14 |
| Ground Cov+HVO | -183.7 | 2.5 | 0.09 |  |  |  |  |
| Shrub Size+GroundCov | -183.8 | 2.6 | 0.09 |  |  |  |  |
| GroundCov+HVO+HVO² | -183.2 | 3.5 | 0.05 |  |  |  |  |
| HVO+HVO² | -185.3 | 5.7 | 0.02 |  |  |  |  |
| Max Ht+HVO+HVO² | -185.2 | 7.7 | 0.01 |  |  |  |  |
| Shrub+HV+ HVO² | -185.3 | 7.8 | 0.01 |  |  |  |  |
| Binary Ht | -188.3 | 9.6 | 0.00 |  |  |  |  |
| Max Ht | -188.6 | 10.0 | 0.00 |  |  |  |  |
| Null | -189.7 | 10.3 | 0.00 |  |  |  |  |
| HVO | -189.1 | 11.1 | 0.00 |  |  |  |  |
| HVO+Max Ht | -188.2 | 11.5 | 0.00 |  |  |  |  |
| Shrub Size+Max Ht | -188.5 | 12.0 | 0.00 |  |  |  |  |
| ***Mus minutoides*** |  |  |  |  |  |  |  |
| HVO + HVO² | -115.7 | 0.0 | 0.35 | HVO* | 6.00 | 1.79 | 2.50–9.51 |
|  |  |  |  | HVO²* | -5.25 | 1.73 | -8.64–-1.85 |
| Shrub+HVO+HVO² | -115.0 | 0.9 | 0.23 | Shrub* | 1.70 | 1.52 | -1.28–4.69 |
|  |  |  |  | HVO* | 5.65 | 1.75 | 2.21–9.08 |
|  |  |  |  | HVO² | -4.73 | 1.75 | -8.15–-1.30 |
| GroundCov+HVO+HVO² | -115.3 | 1.6 | 0.16 | GroundCov | 0.43 | 0.57 | -0.69–1.54 |
|  |  |  |  | HVO* | 5.55 | 1.91 | 1.81–9.29 |
|  |  |  |  | HVO²* | -4.92 | 1.80 | -8.44–-1.38 |
| Max Ht+HV+ HVO² | -115.6 | 2.0 | 0.13 |  |  |  |  |
| GroundCov+MaxHt+HVO+ HVO² | -115.3 | 3.8 | 0.05 |  |  |  |  |
| Shrub Size+GroundCov | -118.7 | 6.1 | 0.02 |  |  |  |  |
| GroundCov | -120.0 | 6.6 | 0.01 |  |  |  |  |
| GroundCov+Max Ht | -119.3 | 7.2 | 0.01 |  |  |  |  |
| Max Ht | -120.7 | 8.0 | 0.01 |  |  |  |  |
| GroundCov+HVO | -119.7 | 8.2 | 0.01 |  |  |  |  |
| Binary Ht | -120.8 | 8.2 | 0.01 |  |  |  |  |
| Shrub Size+Max Ht | -119.9 | 8.6 | 0.01 |  |  |  |  |
| HVO+Max Ht | -120.0 | 8.8 | 0.00 |  |  |  |  |
| HVO | -121.6 | 9.6 | 0.00 |  |  |  |  |
| Null | -123.0 | 10.4 | 0.00 |  |  |  |  |
| ***Mastomys natalensis*** |  |  |  |  |  |  |  |
| Max Ht | -27.5 | 0.0 | 0.27 | MaxHt | -1.13 | 0.58 | -2.27–0.00 |
| Shrub Size+Max Ht | -26.8 | 0.9 | 0.17 | MaxHt | -0.82 | 0.60 | -1.92–-0.29 |
|  |  |  |  | Shrub | 19.54 | 99.57 | -3463–3466 |
| GroundCov+Max Ht | -27.3 | 1.9 | 0.10 | MaxHt | -1.37 | 0.77 | -2.89–0.11 |
|  |  |  |  | GroundCov | 0.34 | 0.59 | -0.81–1.48 |
| HVO+Max Ht | -27.4 | 2.2 | 0.09 |  |  |  |  |
| Max Ht+HVO+HVO² | -26.3 | 2.3 | 0.09 |  |  |  |  |
| Null | -30.0 | 2.8 | 0.06 |  |  |  |  |
| Shrub Size+Ground Cov | -27.8 | 2.9 | 0.06 |  |  |  |  |
| GroundCov+MaxHt+HVO + HVO² | -26.1 | 4.2 | 0.03 |  |  |  |  |
| GroundCov | -29.6 | 4.3 | 0.03 |  |  |  |  |
| HVO | -29.7 | 4.4 | 0.03 |  |  |  |  |
| Binary Ht | -29.7 | 4.6 | 0.03 |  |  |  |  |
| Shrub+HVO+HVO² | -28.0 | 5.5 | 0.02 |  |  |  |  |
| GroundCov+HVO | -29.4 | 6.0 | 0.01 |  |  |  |  |
| GroundCov+HVO+ HVO² | -29.6 | 6.5 | 0.01 |  |  |  |  |
| HVO+HVO² | -29.4 | 8.3 | 0.00 |  |  |  |  |
| ***Dendromus mystacalis*** |  |  |  |  |  |  |  |
| Null | -54.9 | 0.0 | 0.22 |  |  |  |  |
| GroundCov | -53.8 | 0.1 | 0.21 |  |  |  |  |
| Max Ht | -54.5 | 1.5 | 0.11 |  |  |  |  |
| Shrub Size+GroundCov | -53.4 | 1.8 | 0.09 |  |  |  |  |
| HVO | -54.8 | 2.1 | 0.08 |  |  |  |  |
| GroundCov+HVO | -53.6 | 2.2 | 0.07 |  |  |  |  |
| GroundCov+Max Ht | -53.7 | 2.3 | 0.07 |  |  |  |  |
| Shrub Size+Max Ht | -54.3 | 3.6 | 0.04 |  |  |  |  |
| HVO+Max Ht | -54.4 | 3.8 | 0.03 |  |  |  |  |
| HVO+HVO² | -54.8 | 4.5 | 0.02 |  |  |  |  |
| GroundCov+HVO+HVO² | -53.6 | 4.6 | 0.02 |  |  |  |  |
| Max Ht+HVO+HVO² | -54.4 | 6.4 | 0.01 |  |  |  |  |
| Shrub+HVO HVO² | -54.5 | 6.4 | 0.01 |  |  |  |  |
| GroundCov+MaxHt+HVO + HVO² | -53.3 | 6.6 | 0.01 |  |  |  |  |
| Binary Ht | DNC |  |  |  |  |  |  |
| ***Steatomys pratensis*** |  |  |  |  |  |  |  |
| Max Ht | -30.7 | 0.0 | 0.31 | MaxHt | 1.88 | 1.04 | -0.15–3.92 |
| HVO+Max Ht | -29.4 | 0.2 | 0.27 | MaxHt | 1.42 | 0.87 | -0.28–3.12 |
|  |  |  |  | HVO | 0.81 | 0.69 | -0.55–2.16 |
| Shrub Size+Max Ht | -30.2 | 1.9 | 0.12 | MaxHt* | 1.88 | 0.94 | 0.04–3.73 |
|  |  |  |  | Shrub | 1.15 | 1.20 | -1.20–3.49 |
| GroundCov+Max Ht | -30.5 | 2.4 | 0.09 |  |  |  |  |
| Max Ht + HVO+HVO² | -29.5 | 3.4 | 0.06 |  |  |  |  |
| Null | -34.0 | 4.1 | 0.04 |  |  |  |  |
| HVO | -33.2 | 5.0 | 0.03 |  |  |  |  |
| GroundCov+HVO+HVO² | -30.4 | 5.3 | 0.02 |  |  |  |  |
| HVO+HVO² | -32.1 | 5.6 | 0.02 |  |  |  |  |
| GroundCov | -33.5 | 5.7 | 0.02 |  |  |  |  |
| GroundCov+MaxHt+HVO + HVO² | -29.4 | 6.3 | 0.01 |  |  |  |  |
| GroundCov+HVO | -32.5 | 6.4 | 0.01 |  |  |  |  |
| Shrub+HVO+HVO² | -31.5 | 7.4 | 0.01 |  |  |  |  |
| Shrub Size+GroundCov | DNC |  |  |  |  |  |  |
| Binary Ht | DNC |  |  |  |  |  |  |

^a^GroundCov = combined coverage of shrubs, grass, and forbs looking down onto a 1m² circular plot from 1.5 m

HVO = horizontal visual obstruction based on Robel pole (Robel et al. 1970)

HVO² = horizontal visual obstruction based on Robel pole (Robel et al. 1970), squared

Max Ht = height of grass (dm)

Shrub Size = 2 categories of shrub size (2-3 m³ and > 4 m³)

Binary Ht = 2 categories of grass height (> 40 cm and < 40 cm)

DNC = Did no converge
